# Adipose Tissue-Derived Minimally Manipulated Products versus Platelet-Rich Plasma for the Treatment of Knee Osteoarthritis: A Systematic Review of Clinical Evidence and Meta-Analysis

**DOI:** 10.3390/jcm13010067

**Published:** 2023-12-22

**Authors:** Francesca Veronesi, Luca Andriolo, Manuela Salerno, Angelo Boffa, Gianluca Giavaresi, Giuseppe Filardo

**Affiliations:** 1 Surgical Sciences and Technologies, IRCCS Istituto Ortopedico Rizzoli, 40136 Bologna, Italy; francesca.veronesi@ior.it (F.V.); gianluca.giavaresi@ior.it (G.G.); 2 Clinica Ortopedica e Traumatologica 2, IRCCS Istituto Ortopedico Rizzoli, 40136 Bologna, Italy; angelo.boffa@ior.it; 3 Applied and Translational Research (ATR) Center, IRCCS Istituto Ortopedico Rizzoli, 40136 Bologna, Italy; manuela.salerno@ior.it (M.S.);

**Keywords:** adipose tissue, minimally manipulated, micro-fragmented adipose tissue, platelet-rich plasma, knee, osteoarthritis

## Abstract

The use of minimally manipulated adipose tissue (MM-AT) products is gaining increasing interest for the treatment of knee osteoarthritis (OA). MM-AT represents an easy way to exploit adipose tissue properties, although clinical evidence is still limited, as well as their benefits with respect to more documented orthobiologics like platelet-rich plasma (PRP). A systematic review and meta-analysis were performed to evaluate the safety and efficacy of MM-AT products for knee OA management. The risk of bias of the included studies was evaluated using the Dawns and Black checklist for all the included studies and RoB-2.0 for randomized controlled trials (RCTs). Thirty-three clinical studies were included in the qualitative analysis: 13 prospective case series, 10 retrospective case series, 7 RCTs, 2 retrospective comparative studies, and 1 prospective comparative study. An overall clinical improvement and few minor adverse events were observed. Five RCTs comparing MM-AT and PRP injections were meta-analyzed, showing comparable results. The analysis also highlighted the limits of the literature, with only a few high-level trials and an overall low quality. Even though the current literature is still limited, the available evidence suggests the safety and overall positive results of the intra-articular injections of MM-AT products for knee OA treatment.

## 1. Introduction

Osteoarthritis (OA) is a chronic, degenerative, and inflammatory joint pathology representing one of the most disabling diseases and one of the major causes of pain, reducing patient quality of life and affecting daily living activities [1]. OA prevalence is increasing over time due to aging, a higher obesity rate, and more frequent sport-related injuries in the world population [2,3]. Consequently, the number of OA patients requiring treatment is on the rise. Unfortunately, the available therapeutic strategies often fail to alleviate patient symptoms, nor alter OA progression [4,5,6]. This leads patients to require joint replacement surgery, which is not free from risks and should be reserved instead for older patients with advanced stages of OA [7,8]. In this context, research efforts have been made to find new minimally invasive therapeutic solutions for the management of patients affected by OA. Among these, growing interest is increasingly on the development of orthobiologic minimally manipulated disease-modifying therapies, including treatments based on the use of platelet-rich plasma (PRP) and, more recently, mesenchymal stromal cells (MSCs) [9].

MSCs show multi-lineage differentiation, self-renewal, immunomodulatory abilities [10], and trophic activity by releasing growth factors and anti-inflammatory cytokines [11]. MSCs can be harvested from several different tissues and, while bone marrow has been the most commonly used source [12], adipose tissue is gaining increasing interest thanks to its numerous advantages over bone marrow, being harvested with lower discomfort and possessing a higher MSC concentration [13,14,15]. Adipose tissue-derived injectable products demonstrated disease-modifying effects in preclinical studies on OA models, providing objective improvements at both cartilage and synovial membrane levels [16]. In preclinical and clinical scenarios, in vitro culture-expanded MSCs from adipose tissue (ADSCs) have been employed in musculoskeletal diseases [17]. However, their clinical use is complicated by legislation requirements, entail a procedure with two surgical steps (one for cell harvesting and one for injection), take a long time (nearly 2 weeks for culture expansion), and need specialized laboratories (cell factories) [15]. To overcome these issues, several companies have developed medical devices to obtain minimally manipulated adipose tissue (MM-AT) products to be injected into the joint in the same surgical session, in a one-step procedure [18]. These products contain not only ADSCs, but also a heterogeneous group of other cells including pericytes (precursor of MSCs) [19], as well as growth factors, cytokines, and angiogenic factors that concur to enhance their therapeutic potential [20]. MM-AT can represent an easy way to exploit adipose tissue properties for the treatments of OA, although clinical evidence is still limited, as well as their benefits with respect to more documented orthobiologic procedures like PRP [21,22]. 

The aim of this study was to analyse the current literature to understand whether MM-AT injection could represent a safe and effective procedure, with higher clinical potential compared to PRP for the treatment of patients with knee OA. 

## 2. Materials and Methods

### 2.1. Data Source and Searches

**Figure 1:**
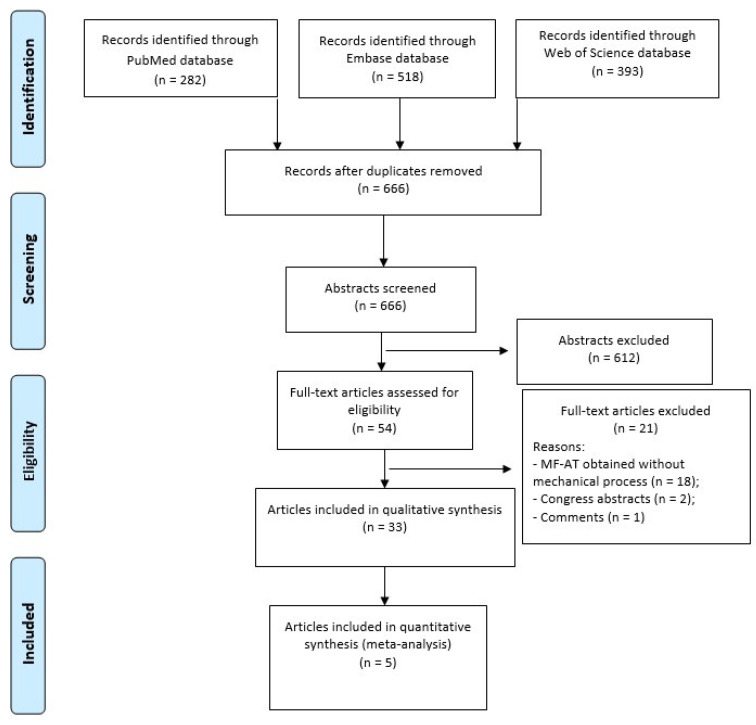
PRISMA flowchart of the study selection process.

A search was performed on 06 June 2023 on the following databases, with no time limits and without any filters: PubMed, Embase, and Web of Science. The following string was applied: (adipose-derived OR micro-fra* adipose tissue OR microfra* adipose tissue OR stromal vascular fraction OR SVF) AND (osteoarthritis) AND (knee). Preferred Reporting Items for Systematic Reviews and Meta-Analyses (PRISMA) guidelines were used and a flowchart of the study selection for qualitative and quantitative data synthesis is reported in Figure 1.

### 2.2. Study Selection

Duplicates were removed through EndNote. Then, the articles were screened by reading the title and abstract according to the following inclusion criteria: clinical studies of any level of evidence, that enrolled more than five patients (case reports with fewer patients were excluded), written in English, on the intra-articular injective treatment with MM-AT for knee joints affected by OA of all grades. Clinical studies with associated treatments, both orthobiologic and surgical ones, were included. Articles were excluded if they were preclinical studies, reviews, book chapters, comments, or technical notes, as well as all procedures that concentrated on adipose tissue with different techniques than mechanical ones, such as those that injected culture-expanded MSCs or stromal vascular fraction (SVF) obtained through enzymatic digestion. Subsequently, the full texts of articles were read in case not enough information could be retrieved from the abstracts, using the inclusion and exclusion criteria listed above. The article selection process was independently performed by two authors (FV and MS) with disagreement on study eligibility solved by a third author (LA).

### 2.3. Data Extraction

Relevant data were independently extracted and collected using a standardized extraction form by two authors (FV and MS). The collected relevant data concerned reference, study type and blinding, type of adipose product and device used, injected amount, ultrasound (US) guidance, associated treatments, control group, patients’ characteristics (number, sex, age, and body mass index—BMI), number of joints, OA grade, final follow-up, and main results.

### 2.4. Risk of Bias

The risk of bias of the studies was assessed independently by two authors (FV and MS) with disagreements resolved by consensus with a third author (AB), using the Dawns and Black checklist [23] for all the included studies and the revised tool for risk of bias in randomized trials (RoB 2.0) [24] approved by the Cochrane collaboration group for the RCTs included in the study.

### 2.5. Quantitative Synthesis and Statistical Analysis

A level I meta-analysis was performed on the RCTs to analyze the outcome of MM-AT injection (treatment group) in comparison with PRP (control group). Statistical analysis and a forest plot were carried out according to Neyeloff et al. using the Meta XL tool for Microsoft Excel. The analysis was carried out using random effects [25] for weighted mean difference of the continuous variables. A statistical test for heterogeneity was first conducted with the Cochran Q statistic and I^2^ metric, which considered the presence of significant heterogeneity with I^2^ values ≥ 25%. When no heterogeneity was found with I^2^ < 25%, a fixed-effect model was used to estimate the expected values and 95% Cis; otherwise, a random-effect model was applied, and an I^2^ metric was evaluated for a random effect to check the correction of heterogeneity. Comparisons among the groups were based on the analysis of variance [26] of the difference between basal and follow-up score (mean difference-MD). All statistical analysis was carried out with Microsoft Excel 2010.

## 3. Results

As shown in Figure 1, the database search identified 1193 records (282 were found in PubMed, 518 in Embase, and 393 in Web of Science). After duplicate removal, the rest of the articles (n = 666) were evaluated by reviewing titles and abstracts according to the inclusion/exclusion criteria, and 54 full-text articles were assessed for eligibility. Among these, 21 articles were excluded because they were congress abstracts (n = 2), editorial comments (n = 1), and about adipose products obtained without mechanical processing (n = 18) with collagenase or other enzymatic digestion. Thus, a total of 33 studies were included in the qualitative analysis and 5 of them in the quantitative synthesis (meta-analysis) (Table 1).

### 3.1. Study Type

**Figure 2:**
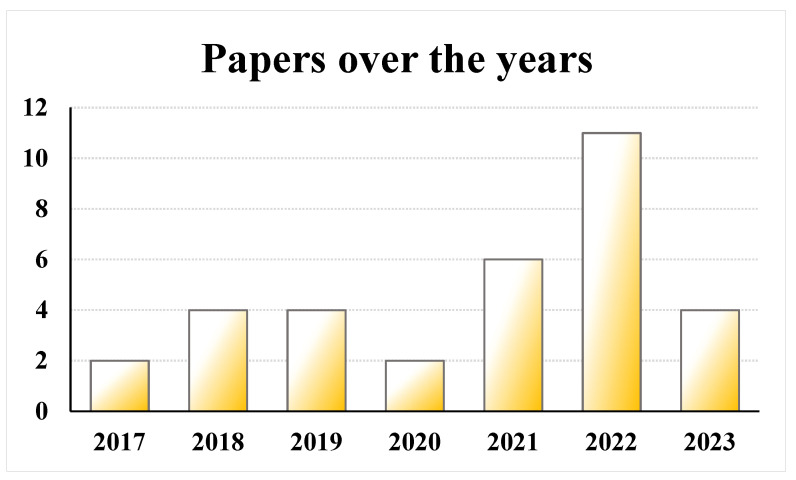
Histograms of the number of studies per year: 2017–2023.

The included studies were published between 2017 and 2023, with a peak in 2022 (n = 11 studies) (Figure 2). Ten studies were comparative trials: prospective RCTs (n = 7), retrospective comparative studies (n = 2), and a prospective comparative study (n = 1). The others were prospective case series (n = 13) and retrospective case series (n = 10). The studies were prevalently not blinded (n = 26); 6 studies were single blinded (n = 6) for the statistician (n = 1), clinical assessor (n = 4), and radiologist (n = 1), and, in one study, the blinding was not reported.

### 3.2. Patient Characteristics

The number of patients treated with only a single intra-articular injection of MM-AT was 1638, while 342 were the patients of control groups. Eighty-one were treated with a combination of surgical and MM-AT injection treatments. Patients lost at the final follow-up were 51 for MM-AT and 23 for control groups. Thirteen studies injected MM-AT bilaterally in some patients [30,32,33,35,36,37,38,41,43,45,46,47,53,54,56,58] and, among them, in one study the treatment was bilateral in all patients [58]. The ratio between males and females was 1.0 in the MM-AT group and 1.4 for the control groups. The patients’ mean age ranged from a mean of 49.0 [28] to a mean of 69.9 [36] years for MM-AT, while 51.9 [29] – 62.5 [32] years was the range for control groups. The mean BMI went from 24.7 [50] to > 35 [40] for MM-AT and from 26.0 [42] to 31.0 [29] for the control groups (12 studies did not report BMI). Patients presented different OA grades, often measured with KL classification: nine studies on KL 1–4 [28,29,39,44,45,46,54,56,59], eight studies on KL 3–4 [30,33,35,40,41,47,48,55], three studies on KL 2–3 [34,42,50], three studies on KL 1–3 [34,44,51], two studies on KL 1–2 [32,37], and two studies on KL 2–4 [36,53]. In addition, patients with mild, moderate, or severe OA grade [43], ICRS grade of 2–4 [51,52], and American College of Rheumatology criteria 1–3 [57] were also evaluated. One study did not report the OA grade [58] and another one gave only the mean KL grade [49].

### 3.3. MM-AT Characteristics and Treatment

All studies harvested adipose tissue from abdominal fat. Almost all studies used a Lipogems (Lipogems International Spa, Milan, Italy) device (n = 29 studies) to process the adipose tissue. In the other four studies, Lipocell (Tiss’You, RSM) [27], Hy-tissue SVF Separation System kit (Fidia, Abano Terme, Italy) [50], MyStemTM kit (MyStem, Wilmington, DE, USA) [53], and Tulip Soft Harvest GOLD System (Tulip Medical, San Diego, CA, USA) [49] were employed, respectively. Only in four studies was the adipose tissue characterized before injection: immunophenotype analysis and viability [29,34,40], total nucleated cell count [29], and histology of lipoaspirate [42] were evaluated. Some studies did not indicate the amount (ml) of injected MM-AT; when reported, the amount was very variable, from a minimum of 3 mL [34] to a maximum of 19 mL [46], with 10 mL being the most commonly injected dose [27,28,30,31,41,44,49,50,51,52,57]. MM-AT was injected through ultrasound (US) guidance in 11 studies [29,33,34,36,38,39,43,44,45,47,56].

MM-AT injection was compared with other orthobiologic treatments, such as PRP (n = 5) [29,32,37,42,59] and BMAC (n = 2) [45,49]. Moreover, the augmentation of intra-articular MM-AT injections to surgical procedures was evaluated in three studies: augmentation to arthroscopic debridement (AD) (n = 2) [48,55] and augmentation to high tibial osteotomy (HTO) (n = 1) [44]. The other studies had no comparison group (n = 23). Few studies (n = 6) evaluated the use of MM-AT products with associated arthroscopic procedures [31,34,35,51,52,57] or other surgical treatments, such as ACL/LCL reconstruction, high tibial osteotomy, and meniscectomy [51,52].

### 3.4. Safety and Complications

As reported in Table 2, 6 studies did not report complications [30,33,42,44,45,48] and, among the other studies, 11 studies did not find complications [27,29,32,39,40,41,46,49,52,54,58]. In the other 16 studies, only one patient had a severe reaction to the injection requiring an arthroscopic wash-out of the joint. The most common complications were related to the harvesting procedure, such as cosmetic changes of the abdominal subcutaneous tissue, abdomen hematoma or pain, swelling/bruising, bleeding, and local erythema, while the others were related to the injective techniques and were localized at the level of the knee joint, such as adipose loose bodies, pain, and effusion, crepitus on motion, and subjective knee instability. In addition, muscle aching in the calves, gallstones, stroke, and tendinopathy were also observed.

### 3.5. Qualitative Analysis

#### 3.5.1. Non-Comparative Studies: MM-AT Injection

Twenty-three studies evaluated MM-AT treatment alone, without comparison groups. The final follow-up was from a minimum of 6 months [27,50] to a maximum of 36 months [52]. Most of the studies had a final follow-up of 12 [28,31,33,34,39,40,41,43,47,51,53,56] months or longer [30,35,36,38,46,54,57,58], and the clinical score used by most of the studies was KOOS (n = 12/23 studies).

All the non-comparative studies reported an improvement in the clinical scores at short-term follow-up, generally also persisting at a longer follow-up [28,30,31,33,34,35,36,38,39,40,41,43,46,47,51,53,54,57]. A decrease in scores at 12 months [56] or at longer a follow-up [58] has been recorded. Three non-comparative studies also analyzed MRI results, reporting an increased GAG content in hyaline cartilage at follow-up [30,41] and a decrease in knee edema [58], without significant cartilage regeneration [58]. Clinical improvement was also reported for advanced OA grade [39,40,47], while negative prognostic factors on the clinical outcome were found to be an age of over 60 and the presence of synovitis [34].

#### 3.5.2. Comparative Studies: MM-AT Injection vs. BMAC Injection

Two non-randomized studies compared the results of a single injection of MM-AT versus BMAC [45,49]. In the first one, a prospective comparative study, after a follow-up of 6 months, a significant improvement of the scores was shown in both groups, without any difference between the two groups. [49] The OA level was found to correlated with the clinical outcome, with patients with KL 2 reporting higher scores than those with KL 3 or 4 [49]. Similarly, in the other study, BMAC and MM-AT showed a significant improvement in scores after a mean of 22 and 13 months of follow-up, respectively, without significant differences between treatments [45].

#### 3.5.3. Comparative Studies: MM-AT Injection Augmentation to Surgical Procedures

Three studies analyzed the effects of the augmentation of intra-articular MM-AT injection to surgical treatments, like HTO [44] or AD [48,55]. The adjunct of an MM-AT injection after HTO significantly improved the clinical outcome in terms of KOOS daily-life assessment score at 12 months of follow-up compared to patients treated with HTO alone. Regarding the augmentation to AD, an RCT analyzed the benefits of intra-articular MM-AT injections after an AD procedure [48,55]. The preliminary results of this RCT on 39 patients did not demonstrate significant difference between patients treated with AD and MM-AT injection versus patients treated with AD alone [48]. However, the final results of this RCT on 78 patients demonstrated better clinical results in terms of KOOS and KSS at 6 months and in terms of KOOS score at 24 months in favor of AD plus MM-AT injections compared to AD alone [55]. Moreover, better T2-mapping scores with magnetic resonance imaging were obtained in the treatment group compared to the control group [55].

### 3.6. Quantitative Analysis: MM-AT vs. PRP

Five RCTs compared a single injection of MM-AT to PRP treatment. Follow-up was at 6 [29], 12 [32,42], and 24 [37,59] months. Regarding complications, two studies did not find AEs [29,32] in both treatment groups, and one study did not report complications [42]. In one study, in the group treated with LP-PRP+HA knee swelling, redness, and mild pain were observed in 48% of cases and synovitis in 8%. In the group treated with MM-AT, donor site ecchymosis and bruising were observed in 20% of the cases [37]. In another study, the MM-AT group presented mild AEs (mild or moderate knee pain and joint swelling and/or effusion) in 18.5% of cases, while pain and edema in the treated knee requiring hospitalization for one day and the use of oral analgesics were observed in one patient. No severe AEs were observed in the PRP group, while knee pain and swelling and/or effusion were present in 11.1% of the cases [59].

The study of Dallo et al. [32] was excluded from the meta-analysis because of overlapping data with the study by Gobbi et al. (follow-up study at longer follow-up) [37].

All the authors of the four included studies employed KOOS subscales and the VAS score to evaluate clinical improvements at 6 months, and three of them employed KOOS subscales and the VAS score for pain at 12 months and the IKDC subjective score at 6 and 12 months.

**Figure 3:**
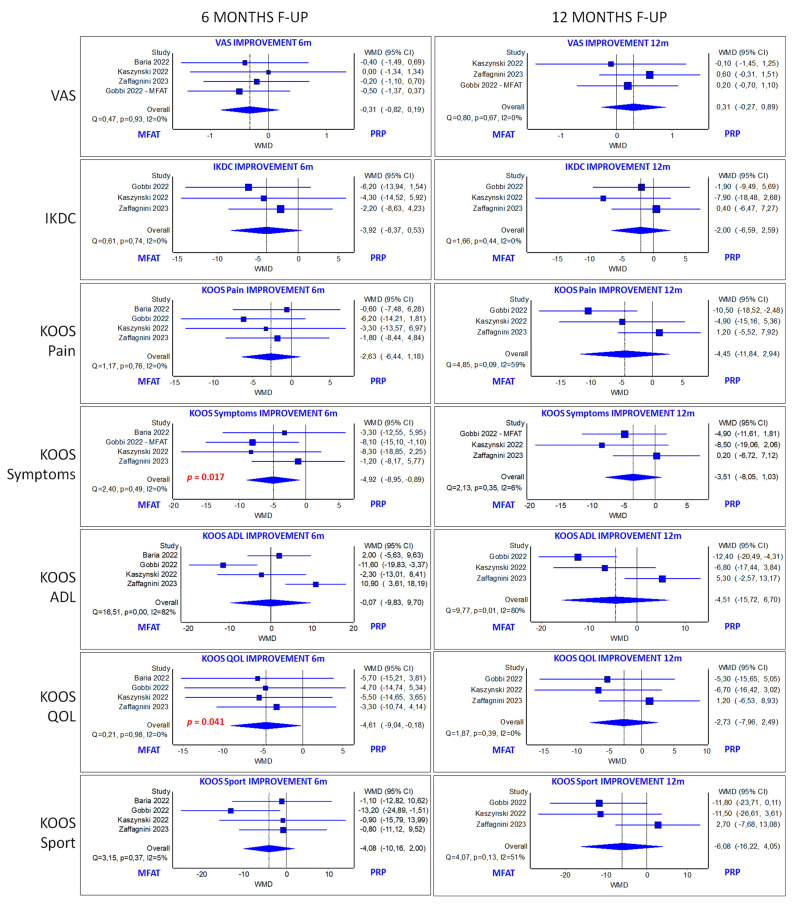
Forest plot of the meta-analyses for visual analog scale (VAS) score, International Knee Documentation Committee (IKDC) score, and Knee Injury and Osteoarthritis Outcome score (KOOS) subscales [29,37,42,59].

Analysis of VAS score did not demonstrate a significant difference between MM-AT and PRP groups at 6 and 12 months of follow-up in terms of improvements from baseline values. Similarly, analysis of the improvement of the subscales KOOS Pain, Koos ADL, and KOOS Sport at 6 and 12 months also did not present a significant difference between the two orthobiologic groups. Conversely, the subscales KOOS Symptoms and KOOS QOL demonstrated a significantly higher improvement in the MM-AT group at 6 months of follow-up (*p* = 0.017 and *p* = 0.041, respectively). However, the mean difference for both subscales (4.9 for KOOS Symptoms and 4.6 for KOOS QOL) did not reach the minimal clinically important difference (8.4 for KOOS Symptoms and 10.3 for KOOS QOL) [60]. Finally, the IKDC subjective score showed a tendency in favor of MM-AT at 6 months but without reaching statistical significance (*p* = 0.084), while no differences were found at 12 months of follow-up. All quantitative analyses are reported in Figure 3.

### 3.7. Risk of Bias

**Figure 4:**
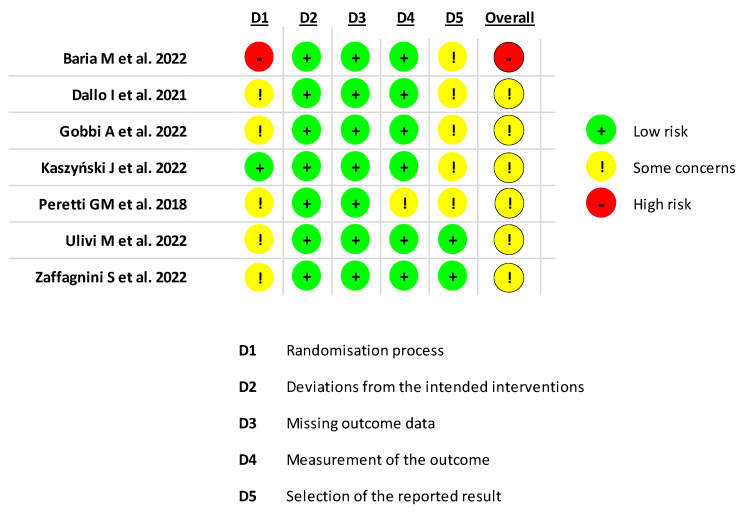
Assessment of risk of bias for randomized controlled trials using the RoB 2.0 tool [29,32,37,42,48,55,59].

A summary of the risk of bias assessment of the RCTs included in the systematic review is illustrated in Figure 4. Six studies had “some concerns” regarding risk of bias [32,36,42,48,55,59] and one study had a high risk of bias [29]. Evaluation with the Downs and Black checklist showed an overall poor quality of the included studies, with an average score of 20.9 (range: 13–26), as reported in Table 3.

## 4. Discussion

The main finding of this systematic review and meta-analysis is that the available clinical evidence suggests the safety and overall positive results of intra-articular injective treatment with MM-AT products for the management of patients affected by knee OA. However, the meta-analysis comparing MM-AT products with PRP injections did not demonstrate the overall superiority of one product over the other. Moreover, the analysis also highlighted the limits of the literature, with only a few high-level trials and an overall low quality of the available clinical studies.

The MM-AT approach is becoming a popular strategy for knee OA to exploit the biological potential of adipose tissue directly as a one-step treatment. This systematic review highlighted a growing interest in this field, with an increasing number of studies published over the years. Nevertheless, clinical evidence is still limited and does not support the large use of these products in orthopedic clinical practice. The quality of the included studies is low, with only seven RCTs published. Among these, five compared MM-AT injections versus PRP treatment. PRP injections present substantial evidence supporting the clinical efficacy in treating patients with knee OA, showing clinically superior benefits compared to placebo as well as to corticosteroids and hyaluronic acid [61]. The overall comparison with PRP did not show the superiority of MM-AT products, except for the subscales KOOS Symptoms and KOOS QOL at 6 months of follow-up. However, for both scores where a difference was detected in favor of MM-AT, this did not overcome the MCID. Accordingly, this difference may not be interpreted as clinically relevant for most patients. In addition, this study reported minor local or systemic complications related to the tissue harvesting procedure and to MM-AT injections. Thus, clinicians should adequately inform their patients about all the possible risks that this therapeutic approach entails. In light of these results and considering the relative invasiveness of the MM-AT approach, the potential side effects, and the higher costs compared with PRP, these adipose-tissue-derived products should not be considered as a first line for the injective treatment of knee OA patients.

In recent years, great interest has been directed towards intra-articular injective treatments for the management of knee OA, aiming to delay or avoid surgery. Several conservative treatments have been proposed to obtain clinical improvement, such as nonsteroidal anti-inflammatory drugs (NSAIDs) or intra-articular injections of corticosteroids or viscosupplementation, which are routinely applied in clinical practice [62]. However, they provide only limited clinical benefits, with low satisfaction often decreasing over time and variable results among patients [62]. This has induced researchers and clinicians to look for new conservative strategies. In this scenario, increasing attention has been turned towards MSC-based therapy, which is potentially able to target the degenerative processes underlying the pathology.

MSCs are considered a biological approach to address articular cartilage pathologies due to their multi-lineage differentiation potential, self-renewal, immunomodulatory capacity, and their ability to release some factors with paracrine effect, which could stimulate cartilage formation by stimulating resident chondrocytes or other cells, angiogenesis, and inhibit joint inflammation [10]. There are several preclinical [63] and clinical [64] studies underlining the potential of in vitro culture-expanded MSCs in OA, showing the potential therapeutic benefit of MSCs for cartilage repair and positive clinical outcomes, with improved joint function, pain level, and quality of life. Despite these positive results, the use of culture-expanded MSCs is limited in clinical practice, considering the presence of strict regulations and the problems related to cell manipulation and expansion with the risk of infection and of allogeneic diseases [15]. Thus, in recent years, minimally manipulated approaches are gaining interest, with the additional advantages of the ease of collection and handling and the minimally invasive procedure required. Treatment with minimal manipulation allows bone marrow or adipose tissue-derived products to be obtained directly in a one-step procedure in the operating room, showing promising results in preclinical and clinical studies performed on OA pathology [65].

Adipose tissue is considered to be an MSC source that is easier to recover and to be handled than bone marrow, which is less affected by aging and which maintains the ability to be differentiated in vitro into osteoblasts, chondrocytes, and adipocytes, according to the different stimuli received [15]. Even though culture-expanded ADSCs and stromal vascular fraction (SVF) are the most studied ways to deliver MSCs and other adipose tissue cells into an affected joint, in recent years great attention has been paid to the use of MM-AT. In preclinical research, MM-AT showed promising results in cartilage repair [21] and induced more synovitis reduction and improvement in articular cartilage status, with no histological differences in comparison to the results offered by SVF and ADSCs [66,67]. MM-AT contains a heterogeneous cell population including fibroblasts, macrophages, adipocytes, endothelial progenitors, pericytes and MSCs, and a low number of leukocytes, which concur to activate anti-inflammatory and pro-regenerative processes [68]. It contains a high number of cells and GFs without requiring culture expansion or enzymatic treatment, thus preserving the integrity of cells and tissue microarchitecture. MM-AT is obtained through lipoaspiration, rinsing with saline to remove blood and oil, and passage through a filter [69]. More in detail, both mechanical and enzymatic methods to process and transfer adipose tissue cells can be employed. However, enzymatic methods give adipose tissue products with higher cell viability and differentiative potential, and destroy the extracellular matrix, which explains why mechanical methods are becoming more attractive and more easily adopted in clinical practice. In addition, mechanical methods induce tissue regeneration stimulation by inducing MSCs to secrete cytokines and angiogenic factors [70]. MM-AT products also have the advantage of preserving the structural properties and integrity of the microarchitecture of the original tissue. In particular, the adipose ‘‘niche’’, which represents the main structural and morphological adipose unit, can be preserved, favoring the therapeutic effects of the residing MSCs [71,72]. Moreover, MM-AT products can reduce friction between cartilage surfaces thanks to their viscosupplementation activity, improving the lubrication of the articular compartment and ultimately alleviating loads on the cartilage surface [20,73].

Thanks to the advantages of MM-AT products, their use in clinical practice is growing for the treatment of patients with knee OA. However, their clinical use is regulated by specific legal requirements which differ among countries and national regulatory agencies. In the United States, ADSCs fall in the category of human cells, tissues, or cellular- and tissue-based products, and currently no such device is approved for clinical use [74]. On the contrary, the European Medicines Agency (EMA) in the European Union defines that non-manipulated tissues are considered as homologous products and approved for clinical application [75]. The potential of cell-based products like MM-AT shows promise to overcome the benefits offered by blood-derived products more broadly applied in clinical practice.

Blood derivatives are orthobiologic treatments used to reduce inflammation and stimulate an anabolic microenvironment. Platelet-derived growth factors, contained in platelets’ alpha granules, together with cytokines, chemokines, and other proteins, possess regenerative, antibacterial, and antifungine properties [76]. PRP is one of the commonly used orthobiologic therapies in orthopedics because of the safe, simple, low-cost, and minimally invasive way to obtain a natural concentration of GFs and bioactive molecules, such as fibroblast growth factor-2 (FGF-2), platelet-derived growth factor (PDGF), transforming growth factor-β (TGF-β), vascular endothelial growth factor (VEGF), and insulin-like growth factor (IGF). It has anti-inflammatory, anti-catabolic, and anabolic properties and induces chondroprotection, supporting its role in the treatment of degenerative conditions such as OA [61,77]. PRP has shown the possibility to delay the progression of OA with promising results, as observed in several preclinical studies, and to offer clinical benefits in clinical trials [29,37,42,61,77]. In a recent meta-analysis, PRP injections provided better results than other injectable options like placebo, hyaluronic acid, and steroids, at both 6 and 12 months after treatment [61]. Accordingly, PRP was used as a reference to measure the potential of MM-AT. To this regard, meta-analysis comparing these two orthobiologics products could be of clinical relevance to understand the most suitable injective option for knee OA patients. Meta-analytic approaches have demonstrated benefits in addressing the limitations of study size, can include diverse populations, and are more valuable than any single study contributing to the analysis [78]. Therefore, the inclusion of high-level trials with the same scores at the same follow-ups allows a comparison to be performed with the highest statistical quality among different treatments.

In the current meta-analysis, the investigation of RCTs comparing the clinical results of MM-AT versus PRP did not confirm the higher potential of a cell-based approach, showing no significant differences between the two groups. Both PRP and MM-AT injections have been investigated as treatment options for knee OA; however, there is no general agreement on their therapeutic efficacy, and no long-term high-level studies are available yet. This, together with the paucity of high-level evidence and heterogeneity in terms of patients included and procedures employed, shows that caution should be taken in drawing conclusions about the potential of MM-AT. These adipose-derived products might exhibit significant variability in terms of cellular composition and growth factor content, based on the employed processing techniques and patients’ characteristics. This heterogeneity could affect clinical responses and consistency of results. Moreover, despite promising theories on their therapeutic potential, clinical results on MM-AT injections are still limited and inconclusive. Therefore, until high-level studies show better results for MM-AT products, this approach should be considered a second-line treatment to address knee OA.

## 5. Conclusions

The available clinical evidence suggests the safety and overall positive results of intra-articular injective treatment with MM-AT products for the management of patients affected by knee OA. However, the analysis also highlights the limits of the literature, with only few high-level trials and an overall low quality of the available clinical studies. Moreover, the meta-analysis comparing MM-AT products with PRP injections does not demonstrate the superiority of one product over the other.

## Figures and Tables

**Table 1:** Characteristics of the clinical studies on MM-AT products for the treatment of knee OA.

Article	Study Type Blinding	Type of Adipose Product (Device)	Injected mL US Guidance	Associated Treatment	Control Group	Pts/Joints	Pts/Joints (Final F-Up)	Age (Mean + SD)	Sex (M/F)	BMI (Mean + SD)	OA Grade	Final F-Up (m)	Main Results
Aletto, 2022 [27]	Prospective case series no	Micro-filtered AT Lipocell (Tiss’You, RSM)	10–15 mL no	no	no	123/123	123/123	57	57/66	27	KL 1–3	6	MM-AT is safe and ameliorates clinical and functional scores in early OA
Barfod, 2019 [28]	Prospective case series no	MM-AT (Lipogems International Spa)	10 mL no	no	no	20/20	20/20	49 ± 9	n.r.	n.r.	n.r.	12	MM-AT is safe and improves functional scores
Baria, 2022 [29]	RCT no	MM-AT (Lipogems International Spa)	7.9 ± 3.9 mL yes	no	LR-PRP	71/71	58/58	51.9 ± 2.4 (LR-PRP); 56.1 ± 1.7 (MM-AT)	28/30	31.0 ± 0.8 (LR-PRP); 31.0 ± 0.9 (MM-AT)	KL 1–4	6	MM-AT and LR-PRP show same clinical improvements
Boric, 2019 [30]	Prospective case series no	MM-AT (Lipogems International Spa)	4–15 mL no	no	no	17/32	10/18	69 ± 12	7/3	n.r.	KL 3–4	24	MM-AT improves GAG content with relevant improvement
Cattaneo, 2018 [31]	Retrospective case series no	MM-AT (Lipogems International Spa)	10 mL no	AS (n: 21) MEN (n: 14)	no	35/35	35/35	53 ± 12 (AS) 55 ± 11 (MEN)	21/14	27 ± 4	KL 1–3	12	MM-AT is safe and adjuvates surgical treatment
Dallo, 2021 [32]	RCT single blind (statistician)	MM-AT (Lipogems International Spa)	n.r. no	no	LP-PRP+HA	50/80	50/80	61.5 ± 9.5 (MM-AT); 62.5 ± 11.3 (LP-PRP+HA)	23/27	26.3 ± 3.6 (LP-PRP+HA); 25.8 ± 5.1 (MM-AT)	KL 1–2	12	LP-PRP+HA and MM-AT show same clinical improvement
Fan, 2023 [33]	Prospective case series no	MM-AT (Lipogems International Spa)	6–8 mL yes	no	no	46/50	46/50	66.9 ± 1.0	28/18	32.0 ± 1.0	KL 3–4	12	MM-AT is safe and effective in moderate-to-severe OA
Ferracini, 2022 [34]	Prospective case series no	MM-AT (Lipogems International Spa)	3–50 mL yes	Arthroscopy	no	101/101	91/91	62.8 ± 10.1	44/47	25.3 ± 3.8	KL 2–3	12	MM-AT, associated with arthroscopy, reduces pain in early/mild OA
Giorgini, 2022 [35]	Retrospective case series no	MM-AT (Lipogems International Spa)	7 mL no	Arthroscopy	no	49/50	45/46	52.7 ± 10.0	24/25	n.r.	KL 3–4	24	MM-AT, associated with arthroscopy, is safe and effective
Gobbi, 2021 [36]	Retrospective case series no	MM-AT (Lipogems International Spa)	5–21 mL yes	no	no	75/120	75/120	Mean 69.6	26/49	Average 28.4	KL 2–4	24	MM-AT improves clinical, functional, and quality of life
Gobbi, 2023 [37]	RCT single blind (clinical assessor)	MM-AT (Lipogems International Spa)	n.r. no	no	LP-PRP+HA	50/80	50/80	62.38 ± 11.88	39/41	n.r.	KL 1–2	24	MM-AT and LP-PRP+HA show same functional improvement, and safety, at mid-term f-up
Heidari, 2021 [38]	Retrospective case series no	MM-AT (Lipogems International Spa)	6–8 mL yes	no	no	220/334	25% pts lost to follow-up	n.r.	125/95	n.r.	KL 3–4	24	MM-AT improves quality of life and can delay TKR
Heidari, 2020 [39]	Prospective case series no	MM-AT (Lipogems International Spa)	6–8 mL yes	no	no	110/110	110/110	42–94	60/50	n.r.	KL 1–4	12	MM-AT improves pain, function, and quality of life
Hudetz, 2019 [40]	Prospective case series no	MM-AT (Lipogems International Spa)	5 mL no	no	no	20/20	17/17	n.r.	15/5	<30 (n: 13); 30–35 (n: 5); >35 (n: 2)	KL 3–4	12	MM-AT shows positive effect in late stages OA
Hudetz, 2017 [41]	Prospective case series no	MM-AT (Lipogems International Spa)	4–15 mL no	no	no	17/32	17/32	69 ± 12	12/5	n.r.	KL 3–4	12	MM-AT improves GAG content, pain, and clinical results
Kaszynski, 2022 [42]	RCT single blind (clinical assessor)	MM-AT (Lipogems International Spa)	n.r. no	no	PRP	54/54	40/40	57 ± 8 (PRP) 55 ± 8 (MM-AT)	n.r.	26 ± 3 (PRP) 27 ± 3 (MM-AT)	KL 2–3	12	MM-AT and PRP show same improvements in pain, symptoms, and functions
Malanga, 2021 [43]	Prospective case series no	MM-AT (Lipogems International Spa)	7.6 ± 2.3 mL yes	no	no	20/23	20/23	59.8 ± 6.5	11/9	28.6 ± 4.8	Mild, Moderate, Severe	12	MM-AT is safe and effective
Magnanelli, 2020 [44]	Retrospective comparative no	MM-AT (Lipogems International Spa)	n.r. no	HTO	HTO	85/85	85/85	n.r.	n.r.	n.r.	KL 1–3	12	MM-AT, associated with HTO, improves the daily life activity
Mautner, 2019 [45]	Retrospective comparative no	MM-AT (Lipogems International Spa)	9 mL yes	no	BMAC	76/106	76/106	59 ± 1 (BMAC); 63 ± 11 (MM-AT)	36/40	n.r.	KL 1–4	21.6 ± 10.6 (BMAC); 13.1 ± 5.9 (MM-AT)	MM-AT and BMAC show same improvement in pain and function
Miles, 2022 [46]	Retrospective case series no	MM-AT (Lipogems International Spa)	3.5–36 mL n.r.	no	no	39/56	37/53	71.1	19/20	28.4	KL 1–4	22	MM-AT improves pain, stiffness, and function
Panchal, 2018 [47]	Prospective case series no	MM-AT (Lipogems International Spa)	n.r. yes	no	no	17/26	17/26	68.27 ± 7.43	10/7	28.98 ± 4.50	KL 3–4	12	MM-AT is safe and effective in refractory severe OA
Peretti, 2018 [48]	RCT (n.r.)	MM-AT (Lipogems International Spa)	19.1 ± 8.1 mL n.r.	AD	AD	39 (of 78 to be included, study ongoing)	16 (8 cases vs. 8 control)	56.25 ± 8.396	70%/30%	n.r.	KL 3–4	6	MM-AT shows encouraging positive trend
Pintore, 2023 [49]	Prospective comparative no	MM-AT (Tulip Soft Harvest GOLD System)	10 mL no	no	BMAC	102/102	102/102	57.64 (BMAC); 61.94 (MM-AT)	46/56	28.76 (BMAC); 26.76 (MM-AT)	KL mean 2.7 (BMAC); KL mean 2.5 (MM-AT)	6	BMAC and MM-AT show same improvement in pain and functions
Priano, 2022 [50]	Retrospective case series no	Non enzymatic SVF (Hy-tissue SVF Separation System kit)	8–10 mL no	no	no	25/25	25/25	53.2 ± 11.7	10/15	24.7 ± 2.1	KL 2–3	6	MM-AT relieves pain and improves stiffness and functions
Russo, 2017 [51]	Retrospective case series no	MM-AT (Lipogems International Spa)	10–15 mL no	ACLR, HTO, MEN (n: 24); arthroscopy (n: 6)	no	30/30	30/30	Median 43	31/9	Median 26	ICRS Grade 2–4	12	MM-AT is safe and feasible in degenerative chondral lesions
Russo, 2018 [52]	Retrospective case series no	MM-AT (Lipogems International Spa)	10–15 mL no	ACLR, HTO, MEN (n: 24); arthroscopy (n: 6)	no	30/30	22/22	Median 43	31/9	Median 26	ICRS Grade 2–4	36	MM-AT is safe in degenerative chondropathy in the mid-term
Santoprete, 2021 [53]	Retrospective case series no	MM-AT (MyStemTM kit)	n.r. no	no	no	84/102	84/102	57.3 ± 4.2	38/46	n.r.	KL ≥ 2	12	MM-AT improves pain, stiffness, and ROM
Screpis, 2022 [54]	Prospective case series no	MM-AT (Lipogems International Spa)	8 mL no	no	no	202/216	202/216	54.0 ± 9.0	97/105	26.8 ± 4.2	KL 1–4	24	MM-AT is safe and effective for symptoms
Ulivi, 2022 [55]	RCT single blind (radiologist)	MM-AT (Lipogems International Spa)	6–8 mL no	AD	AD	78/78	66/66	60.7 ± 7.9	44/34	n.r.	KL 3–4	13–42	MM-AT, associated with AD, improves functions and MRI appearance
Van Genechten, 2021 [56]	Prospective case series no	MM-AT (Lipogems International Spa)	8–10 mL yes	no	no	64	56/77	54.2 ± 9.1	31/33	27.2 ± 4.5	KL 1–4	12	MM-AT shows early clinical improvement but a mediocre response rate
Vasso, 2022 [57]	Retrospective case series no	MM-AT (Lipogems International Spa)	10–15 mL no	AD	no	23/23	23/23	58 ± 8	8/15	28.0 ± 4.8	ACR criteria 1–3	22.1 ± 4.2	MM-AT, associated with AD, improves clinical and functional scores
Yu, 2023 [58]	Prospective case series single blind (clinical assessor)	MM-AT (Lipogems International Spa)	6–8 mL no	no	no	20/40	20/40	54.63 ± 3.90	8/12	25.5 ± 2.86	n.r.	18	MM-AT improves functions and pain, but not in the long term
Zaffagnini, 2022 [59]	RCT single blind (clinical assessor)	MM-AT (Lipogems International Spa)	5 mL no	no	PRP	108/108	108/108	54.5 ± 12.1 (MM-AT); 54.1 ± 10.6 (PRP)	64/44	25.9 ± 4.3 (MM-AT) 28.0 ± 5.5 (PRP)	KL 1–4	24	MM-AT and PRP show same improvements

Abbreviations: ACLR = anterior cruciate ligament reconstruction; ACR = American College of Rheumatology; AD = arthroscopic debridement; AS = arthroscopic shaving; AT = adipose tissue; BMAC = bone marrow concentrate; BMI = body mass index; f-up = follow-up; M = male; F = female; GAG = glycosaminoglycans; HA = hyaluronic acid; HTO = high tibial osteotomy; KL = Kellgren–Lawrence; LR-PRP = leucocyte-rich–platelet-rich plasma; m = months; MM-AT = minimally manipulated adipose tissue; MEN = meniscectomy; MRI = magnetic resonance imaging; OA = osteoarthritis; Pts = patients; RCT = randomized clinical trial; ROM = range of motion; SD = standard deviation; TKR = total knee replacement; US = ultrasound; n.r. = not reported.

**Table 2:** Complications found in each study of the qualitative systematic review. The percentages refer to patients with complications.

Article	Complications Related to Adipose Tissue Harvesting Procedure	Local or Systemic Complications Related to MM-AT Injection
Aletto et al., 2022 [27]	/	/
Barfod et al., 2019 [28]	Cosmetic changes to the abdominal subcutaneous tissue (5%)	/
Baria et al., 2022 [29]	/	/
Boric et al., 2019 [30]	n.r.	n.r.
Cattaneo et al., 2018 [31]	Temporary and small subcutaneous hematoma (2.9%)	/
Dallo et al., 2021 [32]	/	/
Fan et al., 2023 [33]	n.r.	n.r.
Ferracini et al., 2022 [34]	/	Painful adipose loose bodies (2.2%), recurrent episodes of joint effusion (2.2%)
Giorgini et al., 2022 [35]	Hematoma (2.2%)	Knee swelling (8.9%)
Gobbi et al., 2021 [36]	Donor site pain (49%), swelling/bruising (28%)	Knee prolonged swelling (13%)
Gobbi et al., 2023 [37]		
Heidari et al., 2021 [38]	Donor site bleeding (4.1%), pain (6.4%)	Joint swelling and pain (21.8%), severe reaction requiring wash-out of the joint (0.45%)
Heidari et al., 2020 [39]	/	/
Hudetz et al., 2019 [40]	/	/
Hudetz et al., 2017 [41]	/	/
Kaszynski et al., 2022 [42]	n.r.	n.r.
Malanga et al., 2021 [43]	Donor site erythema and swelling (1%), soreness (52.5%), and hematoma (15%)	Knee swelling (15%)
Magnanelli et al., 2020 [44]	n.r.	n.r.
Mautner et al., 2019 [45]	n.r.	n.r.
Miles et al., 2022 [46]	/	/
Panchal et al., 2018 [47]	/	Knee pain and swelling
Peretti et al., 2018 [48]	n.r.	n.r.
Pintore et al., 2023 [49]	/	/
Priano et al., 2022 [50]	/	Knee crepitus on motion (32%) and effusion (4%)
Russo et al., 2017 [51]	Donor site hematoma (6.7%)	Recurrent knee effusions (3.3%)
Russo et al., 2018 [52]	/	/
Santoprete et al., 2021 [53]	Donor site discomfort and pain (15%)	Knee swelling and pain (7%)
Screpis et al., 2022 [54]	/	/
Ulivi et al., 2022 [55]	Donor site small hematoma	/
Van Genechten et al., 2021 [56]	/	Subjective knee instability (3.6%), muscle aching in the calves (1.8%), gallstones (1.8%), stroke (3.6%), tendinopathy (5.4%)
Vasso et al., 2022 [57]	Donor site transitory hematoma (8.7%)	/
Yu et al., 2023 [58]	/	/
Zaffagnini et al., 2022 [59]	/	MM-AT: mild/moderate knee pain, joint swelling, and/or effusion (18.5%); PRP: knee pain, joint swelling, and/or effusion (11.1%)

**Table 3:** Evaluation of the included studies using the Downs and Black checklist.

Article	Reporting	External Validity	Internal Validity Bias	Internal Validity Confounding	Power	Total Score
Aletto C et al., 2022 [27]	7	3	5	3	0	18
Barfod KW et al., 2019 [28]	8	3	5	3	0	19
Baria M et al., 2022 [29]	10	3	5	5	1	24
Boric I et al., 2019 [30]	9	3	4	3	0	19
Mautner K et al., 2019 [45]	8	3	5	4	0	20
Cattaneo G et al., 2018 [31]	10	3	5	3	0	21
Dallo I et al., 2021 [32]	10	3	6	5	1	25
Fan F et al., 2023 [33]	7	3	6	3	0	19
Ferracini R et al., 2022 [34]	11	3	5	4	1	24
Giorgini A et al., 2022 [35]	11	3	5	4	0	23
Gobbi A et al., 2023 [37]	10	3	6	5	1	25
Gobbi A et al., 2021 [36]	10	3	5	3	1	22
Heidari N et al., 2021 [38]	10	3	5	4	0	22
Heidari N et al., 2020 [39]	9	3	5	3	0	20
Hudetz D et al., 2019 [40]	9	3	5	3	0	20
Hudetz D et al., 2017 [41]	10	3	5	3	0	21
Kaszyński J et al., 2022 [42]	9	3	6	4	1	23
Magnanelli M et al., 2020 [44]	4	2	4	3	0	13
Malanga GA et al., 2021 [43]	10	3	5	3	0	21
Miles MR et al., 2022 [46]	11	2	5	4	0	22
Panchal J et al., 2018 [47]	7	3	5	2	0	17
Peretti GM et al., 2018 [48]	7	3	4	3	0	17
Priano V et al., 2022 [50]	9	3	5	3	0	20
Russo A et al., 2017 [51]	10	3	5	3	0	21
Russo A et al., 2018 [52]	9	3	5	3	0	20
Santoprete S et al., 2021 [53]	9	3	4	3	0	19
Screpis D et al., 2022 [54]	11	3	5	4	0	23
Ulivi M et al., 2022 [55]	10	3	5	4	1	23
Van Genechten W et al., 2021 [56]	10	3	5	4	0	22
Vasso M et al., 2022 [57]	11	2	5	4	0	22
Zaffagnini S et al., 2022 [59]	11	3	6	5	1	26
Pintore A et al., 2023 [49]	10	2	4	3	0	19
Yu Y et al., 2023 [58]	9	2	6	3	1	21

## Data Availability

The data that support the findings of this study are available from the corresponding author upon reasonable request.
